# Understanding the Environmental Background of an Invasive Plant Species (*Asclepias syriaca*) for the Future: An Application of LUCAS Field Photographs and Machine Learning Algorithm Methods

**DOI:** 10.3390/plants8120593

**Published:** 2019-12-12

**Authors:** Péter Szilassi, Gábor Szatmári, László Pásztor, Mátyás Árvai, József Szatmári, Katalin Szitár, Levente Papp

**Affiliations:** 1 Department of Physical Geography and Geoinformatics, University of Szeged, Egyetem utca 2, 6722 Szeged, Hungary; szatmari@geo.u-szeged.hu; 2 Institute for Soil Sciences and Agricultural Chemistry, Centre for Agricultural Research, Herman Ottó út 15, 1022 Budapest, Hungary; szatmari@rissac.hu (G.S.); pasztor@rissac.hu (L.P.); arvai.matyas@rissac.hu (M.Á.); 3 Institute of Ecology and Botany, Centre for Ecological Research, Alkotmány u. 2-4, 2163 Vácrátót, Hungary; katalin.szitar@gmail.com; 4 Department of Geoinformatics, University of Salzburg, Schillerstraße 30, 5020 Salzburg, Austria; papplevente9610@gmail.com

**Keywords:** CORINE, Common milkweed, land cover change, soil texture, aridification, potential infected area, environmental driving forces, biological invasions

## Abstract

For developing global strategies against the dramatic spread of invasive species, we need to identify the geographical, environmental, and socioeconomic factors determining the spatial distribution of invasive species. In our study, we investigated these factors influencing the occurrences of common milkweed (*Asclepias syriaca* L.), an invasive plant species that is of great concern to the European Union (EU). In a Hungarian study area, we used country-scale soil and climate databases, as well as an EU-scale land cover databases (CORINE) for the analyses. For the abundance data of *A. syriaca*, we applied the field survey photos from the Land Use and Coverage Area Frame Survey (LUCAS) Land Cover database for the European Union. With machine learning algorithm methods, we quantified the relative weight of the environmental variables on the abundance of common milkweed. According to our findings, soil texture and soil type (sandy soils) were the most important variables determining the occurrence of this species. We could exactly identify the actual land cover types and the recent land cover changes that have a significant role in the occurrence the common milkweed in Europe. We could also show the role of climatic conditions of the study area in the occurrence of this species, and we could prepare the potential distribution map of common milkweed for the study area.

## 1. Introduction

Biological invasion is considered to be one of the largest threats to native biodiversity [1,2,3]. Increasing eradication efforts have to be made to keep up with the growing rate of introduction and establishment of alien species [4]. Because of limited financial resources, global strategies need to identify and implement optimal management procedures against the spread of invasive species [5]. Previous studies emphasize that prevention and early detection with rapid response are the most efficient ways to combat invasion [2,5]. Beside early detection, the monitoring of existing populations of invasive species is also part of an effective invasion control strategy [6]. Early detection should be based on a system of regular surveys to find newly established populations [7]; however, most of these studies lack European and national scales [8]. Applicable methods to detect species may differ between taxonomic groups, population size, and environmental settings [9]. Remote sensing data, including aerial photographs and satellite images, have been widely used to map invaders; however, they are most effective for species appearing in and dominating the canopy of the ecosystem [4], and when the population size is rather high [10].

Conspicuous, easily identifiable alien plant species may be detected by the use of field photographs. However, field photos are usually taken by different individuals in an unrepresentative way in space and time, or with small spatial coverage. Statistical Office of the European Communities (EUROSTAT) has been carrying out the Land Use and Coverage Area Frame Survey (LUCAS) every three years since 2006, in order to identify the actual status and changes in land use and cover in the European Union (EU) [11]. This survey was established for the validation of the Coordination of Information on the Environment (CORINE) land cover (CLC) database—therefore, it is carried out in situ. LUCAS points belong to the intersections of a 2 km grid that includes around 1 million points all over the EU. As part of the LUCAS procedure, field images are taken at the survey points that can be used for assessing the presence of invasive species. The density and the spatial extent of the survey points allows for the systematic assessment of invasive species presence at regional, national, and EU-wide spatial scales [12].

According to our hypothesis, the field photographs of the EUROSTAT LUCAS database can be used for mapping the potentially infected area of common milkweed or other invasive plant species. We also supposed that the machine learning algorithm could be a very useful statistical tool for identifying the main environmental driving forces of spreading of plant invasions.

In relation to invasive species, a major goal is to predict where new populations are likely to emerge. Beside anthropogenic dispersal, abiotic drivers shape the large-scale distribution of invasive species. Land use change is among the main determinants of biological invasion [13,14,15,16,17,18]. The invasion potential of an invasive species can be determined by surveying a wide range of environments for established populations [6]. The identification of land use and other abiotic, geographical characters of the sites already invaded by invasive species can reveal potential locations suitable for new emergence [7,19], which should be the focus points of early detection and rapid eradication.

Common milkweed (*Asclepias syriaca* L.) is an invasive perennial herb species of North American origin. After recognizing the ongoing secondary range expansion, as well as the economic and ecological importance of common milkweed, it was included in the list of invasive alien species of European Union concern (EU Regulation 1143/2014). It has already been naturalized in 23 countries worldwide, and anticipated climate changes are predicted to help further its future spread beyond its current distribution [20].

Common milkweed was introduced into Europe in 1629 [21]. In the invaded range, the species occurs mainly in ruderal habitats, road verges [22], abandoned fields, and tree plantations [21]. The study of its ecological effects has been gaining importance given its range expansion in recent decades. Invasion of common milkweed has been linked with decreased levels of native plant species cover [23], shifts of maximum vegetation compositional diversity to larger spatial scales in sand grasslands [24], and lower levels of the functional diversity of spiders [25] in invaded ecosystems. In contrast, Jurová et al. [26] did not find any negative effects of common milkweed in soil nematode community, while Szitár et al. [27] showed positive effects of common milkweed on native grass survival during summer drought.

In our study, we used LUCAS survey field photos for the Southern Great Plain (nomenclature of territorial units for statistics (NUTS) level 2 statistical region) in Hungary, in order to assess the occurrence of common milkweed populations and identified environmental factors driving milkweed invasion.

Our specific research questions were as follows:

- How can we identify the biogeographical and anthropogenic factors involved in the appearance of an invasive plant species (such as common milkweed)?
- What geological, pedological, and land cover factors influence the appearance of common milkweed?
- What significance do environmental (biogeographical) factors and land cover have in the presence of common milkweed? What kind of relationship characterizes the system?
- How can the area potentially infected with common milkweed be outlined within the research area?

Based on the quantified responses to the above questions, it will be possible to prepare a map showing the potential spread of common milkweed on a regional (continental) scale.

## 2. Results and Discussion

Table 1 presents the confusion matrix of the validation, based on the test set consisting of 243 LUCAS observations, which were independent from the whole modeling process. Based on the confusion matrix the accuracy of the model—i.e., the sum of the true positives and true negatives divided by the total population—was equal to 0.843, which can be regarded as promising accuracy. However, the false negatives, also known as type II errors, were 28, which can be explained by the fact that the observations used in this study are unbalanced to the absence of milkweed.

When we study the rank and weight of factors affecting the presence of common milkweed, it can be seen that the physical characteristic of soils is the primary factor determining the appearance of this species. Academic literature also indicates that loose, less cemented soils are favored by this species [12,21,28]. According to our results, there is 100% correlation between soil texture and the appearance of common milkweed (Table 2).

In addition to physical properties, the type of soil and the current land cover of a given area are the factors that significantly influence the presence of common milkweed with a weight above 10%. Land cover changes are also important factors, as well as the CaCO_3_ content, the pH of upper soils, and the climatic conditions of the area (annual rainfall, mean temperature, evaporation).

Thus, climatic factors do influence the appearance of common milkweed, though they are not dominant factors. According to the climate scenarios of the sample area, summer precipitation is likely to decrease with the increase in the mean annual temperature, which predicts an increase in the annual evaporation [29,30]. We can also verify the findings of [31], in which the authors suppose that suboptimal climatic conditions (precipitation, evaporation, and temperature levels) may limit the northward expansion of the species in Europe.

Most of the characteristics and conditions, then, correspond to rules that affect the current occurrence and the future spread of common milkweed relate to soil characteristics (10 out of 13) and land cover and land cover changes (6 out of 13) of the sample area (Table A1). Soil type and physical properties are the most important pedological factors influencing the appearance of the species (See Table A1, Rules 1 and 13). According to academic literature, loose sandy soils are favored by this plant, because it can grow its rhizomes deep to the level of groundwater [21]. Humic sandy soils, sandy loam, and loamy sand soil texture appear in most of the rules. In addition, slightly alkaline soils with a pH of 7.4–8.5 and a CaCO_3_ content higher than 14.5% are favored by this plant. The current type of land cover is also an important factor for this species. Among the land cover categories of the 2012 CLC database, common milkweed prefers areas with complex cultivation patterns and natural grassland areas. The latter factor is of particular concern, because the natural grasslands that are native to the area have significant conservation value [27]. Common milkweed has been primarily invading disturbed areas, such as abandoned agricultural lands [21]. This alien species prefers sandy soils with very low water absorption, high water conductivity, poor water storage capacity, and very low water retention properties [12]. We found that, due to the current land cover, the appearance of common milkweed is also affected by land cover changes. We identified the types of land cover changes that promote the appearance of common milkweed.

**Figure 1:**
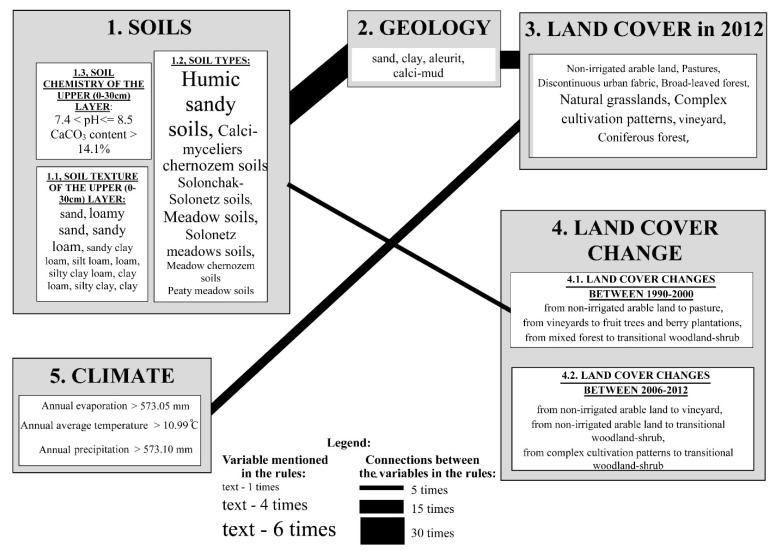
The weight of variables affecting the presence of common milkweed and those variables’ relationship to one another (based on the rules of the machine learning algorithm).

According to our results, most of the geographical and environmental factors do not influence the appearance of common milkweed independently, but in relation to one another (Figure 1). Soil properties and geological features usually appear in the same rules, so their joint influence is predominant in the spread of common milkweed (Table A1). Changes in soil properties and land cover are also connected with each other, leading to an explosive increase in the amount of areas infected by common milkweed since the 1990s (Figure 1).

**Figure 2:**
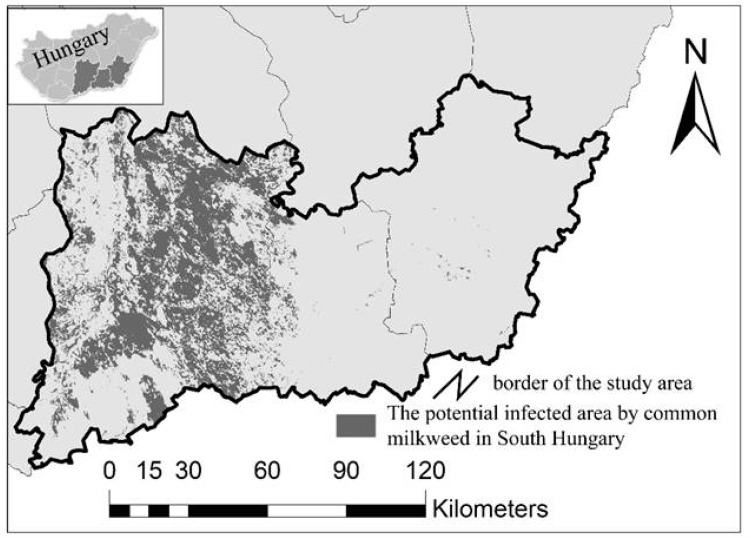
Areas potentially infected with common milkweed within the study area (based on the results of machine learning algorithm).

By employing the machine learning algorithm method, we could create a map depicting the potential spread of common milkweed, especially in the western part of the study area, due to its favorable geographical and environmental conditions. These areas are dominated by sandy soils, and their land cover changes, as well as current land cover, are preferred by common milkweed (Figure 2).

## 3. Materials and Methods

### 3.1. Study Area

Our study area is one of the areas that is most heavily invaded by *A. syriaca* within Hungary [21]. The research area is the Southern Great Plain Region, which is one of the NUTS 2 (nomenclature of territorial units for statistics; basic regions for the application of regional policies) statistical regions of Hungary. The largest continuous sandy area in Central Europe is located in the western part of the study area, which is characterized by poor soil fertility. The area on the eastern border of the study area are loess plains with chernozem soil, which are more favorable for agriculture. Due to its favorable physical geographical features, the Southern Great Plain is a cultural landscape characterized by agricultural land use that is adapted to the characteristic physical geographical features (soil, hydrology, and climate) [23,29]. Due to the physical geographical features of sandy areas, diverse land use (forest, grassland, arable land, vineyards, and orchards) has developed [32]. The natural values of the Southern Great Plain region (including sandy grasslands) are protected by the Kiskunság National Park.

The sample area has a humid continental climate, with an average annual temperature of 10.5 °C and an average annual precipitation of 550 mm. Agriculture is facing major challenges as a result of its more and more arid climate, which is characterized by diminishing summer rainfall, increasing drought frequency, and the significant decline in groundwater level (up to 6–8 m in some areas) since 1970 [16,29,33,34]. Since the 1990s, the main land use changes of these areas have been arable land abandonment and the conversion of grassland to plantation. The abandonment of arable land was most significant in sandy areas, which are very sensitive to drought and have poor water retention capabilities [16,29,33,34].

### 3.2. Identification of Sites Invaded by Common Milkweed

The LUCAS data collection includes the preparation of field photos at each observation point; the coordinates of each of these points were preliminarily defined according to a standardized sampling design over the whole territory of the EU LUCAS 2009, 2012, and 2015 databases [35,36,37,38]. We used 3892 field photos of 973 LUCAS survey points from 2015, which are located within the study area and surveyed during spring (April and May).

**Figure A1:**
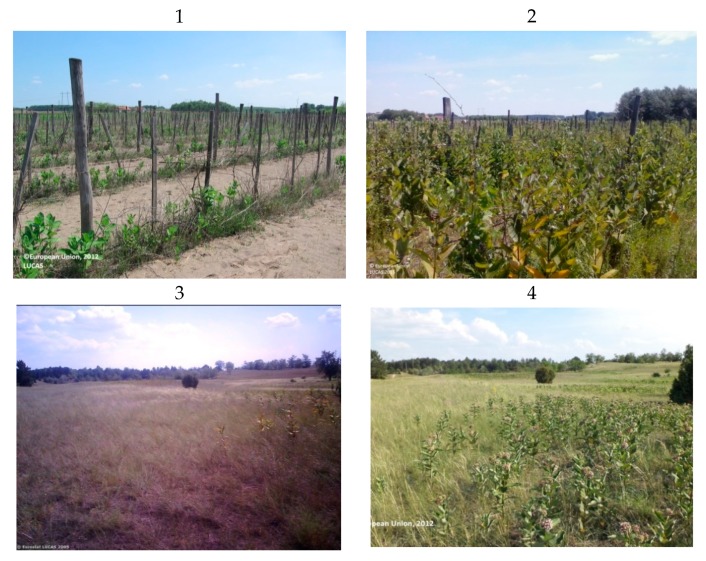

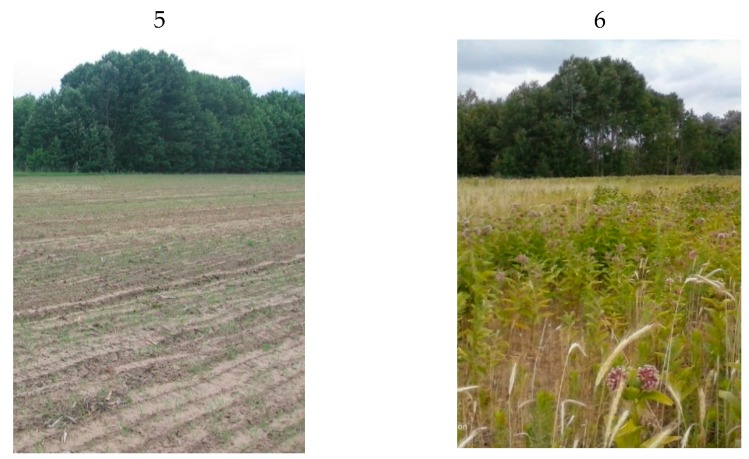
Photo pairs of the same LUCAS observation points which shows the dramatic invasion of *Asclepias syriaca*: 1–2, Abandoned vineyard in 2012 and in 2015, 3–4, Grassland in 2009 and in 2012, 5–6, Arable land in 2009 and in 2012. Data source: LUCAS photos.

The landscape photographs from the field survey points were evaluated by visual interpretation. It was easy to identify the individuals or the homogenous patches of common milkweed in the field photos (Figure A1) because of the characteristic morphological features of the plants (e.g., bright and shiny green leaves and occasionally purple inflorescences). Based on the presence of common milkweed in at least one of the four photographs taken at each point, survey points were classified as 0 (no common milkweed present) and 1 (common milkweed is present; site is invaded by common milkweed) [12].

### 3.3. Digital Databases for Investigating the Environmental Driving Forces of Common Milkweed Spreading

We used various environmental covariates for identifying the geographical driving forces that may determine the occurrence of common milkweed (Table 3). They were classified into five classes, depending on their roles in landscape ecology. The first class was soil, which quantifies the productivity of a habitat. The second class was topography, in which covariates were selected that provided information on the vertical and horizontal distance to the channel network. These were derived from the SRTM (Shuttle Radar Topography Mission) 100-meter-resolution digital elevation model [39]. The third one was land cover, which was represented by the CORINE Land Cover survey products from 2012 and by the land cover change between 1990–2000, 2000–2006, and 2006–2012. The fourth class was climate, which was characterized by the most common climatic parameters. The last was geology, where the parent material was considered as a potential environmental covariate.

### 3.4. Statistical Methods

#### 3.4.1. Exploratory Analysis and Pre-Processing of Data

**Figure 3:**
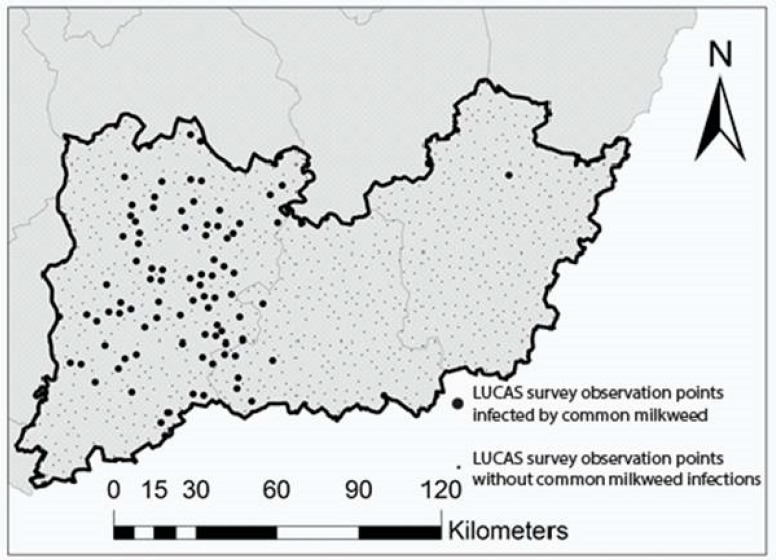
The spatial distribution of common milkweed infection in the study area, based on the visual analyses of the LUCAS field survey photos.

The classified database containing 973 LUCAS survey points on common milkweed was converted into a dummy variable. The created dummy variable makes it possible to explore the potential geographical driving forces and factors of common milkweed spreading. After binary coding, we observed that the dummy variable had unbalanced data, i.e., the dummy value 0 (no milkweed) was overrepresented at the expense of the dummy value 1 (milkweed present). The proportions were 88% and 12% for the dummy values 0 and 1, respectively (Figure 3).

This unbalance must be handled before one can apply any statistical techniques, because a number of statistical analyses, including machine learning techniques, are sensitive to unbalanced data. For this purpose, various techniques could be applied, including up-sampling, down-sampling or a combination of both. In the present study, we used Random Over-Sampling Examples (ROSE) [48], which is a combination of up- and down-sampling techniques.

We split the data into a training set and a test set, where the training set was used to explore and model the relationship between the dummy variable and the environmental covariates, whereas the test set was used to validate the resulting model. The data partition was carried out randomly, with the addition that it had to take into consideration the unbalanced nature of the data—i.e., both the training and test sets had to honor the initial proportion of the absence and presence of common milkweed. From there, 75% of the data (i.e., 730 observations) went to the training set, while the remaining 25% (i.e., 243 observations) went to the test set, which was independent of the whole modeling process.

#### 3.4.2. The C5.0 Classification Model

The hypothesis was that a machine learning algorithm (MLA) could be applied, because MLAs commonly outperform classical statistical techniques (e.g., linear regression, generalized linear model, logistic regression) by stressing prediction accuracy [49]. However, one of the main drawbacks of MLAs is that the results are not as easy to interpret as those of classical techniques [49]. Therefore, we searched for an MLA that would provide more or less interpretable results, because our aim was not just to explore and model the relationship between common milkweed spreading and its geographical driving factors, but also to predict further sites in the region with high invasion potential. Thus, we chose the C5.0 classification model [50]. Briefly, the C5.0 classification model is an MLA resulting in either decision trees or sets of if–then rules (so-called “rulesets”) using information gain (entropy) as a splitting criterion. In addition, C5.0 is quite a robust technique, considering multicollinearity. Compared to more advanced and sophisticated MLAs (e.g., neural networks, support vector machines, and random forests), the decision trees or sets of if–then rules under C5.0 generally perform nearly as well, but are much easier to understand and deploy. For more details, see [50,51].

We fine-tuned the C5.0 model by setting up a grid of tuning parameters using the train function of the R package “caret” [52]. Five-fold repeated cross-validation was used during the fine-tuning. The aforementioned unbalanced data were handled by the R package ROSE [38], which down-samples the majority class and synthesizes new data points in the minority class. The selected quality metric was the ROC (receiver operating characteristic). The best tune of the C5.0 model was validated using the test set, and it was used (i) for exploring and interpreting the geographical driving forces of common milkweed occurrence, and (ii) for mapping the potential occurrence of common milkweed using the spatially exhaustive environmental covariates listed in Table 3.

## 4. Conclusions

According to our results, the appearance of common milkweed is strongly related to certain soil types (e.g., humic sandy soils), as well as certain physical, chemical soil properties (e.g., soil texture sandy loam soils, pH between 7.4 and 8.5, CaCO_3_ content less than 14.1%), current land use type (e.g., natural grassland, or complex cultivation pattern), and certain types of land use change (e.g., land abandonment from arable lands into pastures). Our statistical analyses proved that this species is common in abandoned arable land, deforested clearings, and other areas with varying land cover. We could show that climate change also plays a role in the appearance of this species. In addition to increasing annual precipitation, evaporation and an increase in average annual temperatures (i.e., drier and drier summers) are particularly favorable for the presence of this species. The presented results provide important contributions for the future modeling of the spread of an invasive species, which in this case is spreading in 23 European countries at a significant rate, and to map its potential occurrence on a regional scale. The machine learning algorithm used in this study is not only suitable for identifying the environmental factors responsible for the appearance of a particular invasive plant, but we were also able to map the areas that could be potentially infected with common milkweed.

## Figures and Tables

**Table 1:** Confusion matrix based on the test set consisting of 243 independent observations on milkweed presence/absence.

	Absence (Observed)	Presence (Observed)
Absence (predicted)	186	10
Presence (predicted)	28	19

**Table 2:** Variables explaining the presence of common milkweed and its relative weight, according to the results of machine learning algorithm methods.

Significance Level of a Given Geographical Condition (Variables)	Name of the Geographical Conditions (Variables)
100.00%	Soil texture of the upper soil layer (between 0–30 cm)
11.82%	Soil types
11.11%	Land cover in 2012
8.29%	Geology
7.58%	Land cover change between 1990–2000
7.05%	Land cover change between 2006–2012
3.70%	CaCO_3_ content of the upper soil layer (between 0–30 cm)
3.70%	pH of the upper soil layer (between 0–30 cm)
2.82%	Annual precipitation
2.65%	Annual average temperature
1.59%	Annual evaporation

**Table 3:** Summary of the applied environmental covariates.

	Name	Reference	Type
*Soil*	Soil type	[40]	Categorical
	United States Department of Agriculture (USDA) soil texture (0–30 cm)	[41]	Categorical
	Calcium carbonate (0–30 cm) [%]	[40]	Continuous
	pH (0–30 cm)	[40]	Continuous
	Sand content (0–30 cm) [%]	[42]	Continuous
	Clay content (0–30 cm) [%]	[42]	Continuous
	Silt content (0–30 cm) [%]	[42]	Continuous
	Organic matter content (0–30 cm) [%]	[42,43]	Continuous
	Rooting depth [m]	[40]	Continuous
*Topography*	Groundwater level [m]	[44]	Continuous
	Vertical distance to channel network [m]	derived from a digital elevation model	Continuous
	Distance to channel network [m]	derived from a digital elevation model	Continuous
*Land cover*	CORINE Land Cover (2012)	[45]	Categorical
	CORINE Land Cover (1990–2000)	[45]	Categorical
	CORINE Land Cover (2000–2006)	[45]	Categorical
	CORINE Land Cover (2006–2012)	[45]	Categorical
*Climate*	Long-term mean annual precipitation	[46]	Continuous
	Long-term mean annual temperature	[46]	Continuous
	Long-term mean annual evaporation	[46]	Continuous
	Long-term mean annual evapotranspiration	[46]	Continuous
*Geology*	Parent material	[44,47]	Categorical

## Appendix A

**Table A1:** The biogeographical and environmental rules of common milkweed (based on the results of machine learning algorithm).

Rules number	1. SOILS	2. GEOLOGY	3. LAND COVER in 2012	4. LAND COVER CHANGE	5. CLIMATE
1	SOIL TYPES: Humic sandy soils, Calci-myceliers chernozem soils, Solonchak-Solonetz soils, Meadow soils	Sand	Non-irrigated arable land	no change between 2006–2012	-
2	SOIL TYPES: Humic sandy soils, Solonchak-Solonetz soils, Solonetz-meadow soils	-	Pastures	no change between 1990–2000 no change between 2006–2012	-
3	SOIL TYPES: Humic sandy soils, Solonetz-meadow soils, Meadow soils	-	Discontinuous urban fabric, Broad-leaved forest, Natural grasslands	no change between 1990–2000	-
4	SOIL TEXTURE OF THE UPPER (0–30 cm) LAYER: sand, loamy sand, sandy loam SOIL TYPES Humic sandy soils, Calci-myceliers chernozem soils, Solonetz-meadow soils	-	-	no change between 1990–2000 no change 2006–2012	Annual rainfall is over 537.1 mm
5	SOIL TYPES: Humic sandy soils, Solonchak-Solonetz soils, Meadow soils	-	-	Land cover change between 1990–2000: from Non-irrigated arable land to pasture, from Vineyards to Fruit trees and berry plantations, from Mixed forest to Transitional woodland-shrub	-
6	SOIL TEXTURE OF THE UPPER (0–30 cm) LAYER: loamy sand, sandy loam SOIL TYPES: Chernozem medow soils, Peaty meadow soils	-	-	-	-
7	-	-	-	Land cover change between 2006–2012 from Non-irrigated arable land to vineyard, from from Non-irrigated arable land to Transitional woodland-shrub, from Complex cultivation patterns to Transitional woodland-shrub,	-
8	SOIL TYPES: Humic sandy soils, Calci-myceliers chernozem soils, Meadow soils	clay, aleurit, calci-mud	Complex cultivation patterns	-	-
9	SOIL TEXTURE OF THE UPPER (0–30 cm) LAYER: sandy clay loam, silt loam, loam, silty clay loam, clay loam, silty clay, clay	-	-	-	-
10	-		Vineyard, Complex cultivation patterns, Coniferous forest, Natural grasslands	-	Annual average temperature is over 10.9 °C
11	-		Vineyard, Complex cultivation patterns, Coniferous forest, Natural grasslands	-	Annual evaporation is over 573 mm
12	SOIL CHEMISTRY OF THE UPPER (0–30 cm) LAYER: 7.4 < soil ph <= 8.5 CaCO_3_ content > 14.1% SOIL TEXTURE OF THE UPPER (0–30 cm) LAYER:silt loam, loam	clay, aleurit, calci-mud		-	-
13	SOIL TEXTURE OF THE UPPER (0–30 cm) LAYER: sand, loamy sand, sandy loam	-	-	-	
